# Supplementary material for: Nanoparticle-Based Radioconjugates for Targeted Imaging and Therapy of Prostate Cancer
Source: Molecules. 2023 May 16;28(10):4122. doi: 10.3390/molecules28104122 (PMC10223532; doi:10.3390/molecules28104122)
Supplement: Supplementary file 1 [file molecules-28-04122-s001.zip › molecules-2339319-supplementary.pdf]

Table S1. Summary of outcomes of *in vitro* and *in vivo* studies included in this review.

NPs	In vitro and in-vivo outcomes	Reference
gadolinium vanadate NPs (GdVO4)	specific targeting ability, clear visualization of tumour by PET, strong fluorescence and narrow emission band, excellent spatial resolution in MRI, rapid accumulation in the liver and spleen, followed by slow degradation.	[46]
micellar NPs (LNP)	specific targeting ability, clear visualization of tumour by PET, rapid accumulation in the liver, kidney, spleen, bladder and tumour, followed by slow degradation, clear accumulation in the tumour with residual amounts in the liver 2 days after injection.	[42]
copper sulfide NPs (CuS)	specific targeting ability, clear visualization of tumour by PET, high accumulation in the liver and bladder, high accumulation and fast clearance in lung tissue, gradual accumulation in the tumour, no acute toxicity.	[45]
iron oxide NPs (IO)	specific targeting ability, MR images showed a high uptake by tumour with high resolution, but were limited in providing quantitative information, PET images showed lower resolution, but provided quantitative information.	[47]
iron oxide NPs (mNP-S1/2) (mNP-N1/2)	specific targeting ability, low toxicity, high avidity and internalization rates *in vitro*.	[48]
quantum dots (QDs)	specific targeting ability, clear visualization of slowly increased accumulation in the tumour, moderate accumulation in the kidney and urinary bladder and high accumulation in the liver, followed by fast clearance in the kidneys, liver, and urinary bladder by PET and NIFR, no acute toxicity.	[49]
melanin NPs (MNPs)	specific targeting ability, no toxicity *in vitro* and *in vivo*, clear visualization of tumour by PET, accumulation in the heart, liver, spleen, intestine and tumour at 2 h after injection, gradually increased accumulation in the tumour, long retention in the tumour.	[44]
polymer PEG NPs (PEG-(DFB)1) (PEG-(DFB)3)	specific targeting ability, clear visualization of tumour by PET, enhanced tumour/blood ratio and background clearance by multivalent targeting ligands, , enhanced tumour accumulation by single valent targeting ligand.	[40]
micellar NPs (CCPM)	specific targeting ability, clear visualization of tumour by both SPECT and NIFR *in vivo*, clear intra-tumoral visualization by optical imaging, longer circulation of NPs-based radioconjugate in the blood than targeting ligand, high accumulation in the liver and spleen, moderate accumulation in the kidney, lung and tumour.	[41]
poly(lactic acid)−polyethyene glycol NPs (PLA−PEG)	no specific targeting ability, high accumulation in the spleen, liver and intestine, similar accumulation of targeted NPs in PSMA(+) and PSMA(−) tumours, similar accumulation of targeted and untargeted NPs in almost all tissues except for liver and tumour, moderately more rapid clearance from the PSMA(+) tumours of the untargeted NPs relative to the targeted NPs in vivo, localization of NPs at the rim of both tumour types,	[39]
gold NPs (AuNP)	specific targeting ability, evident tumour uptake at 1 h, high accumulation in the liver, pancreas and spleen, possibility to acquire the transaxial, coronal and sagittal SPECT images,	[31]
quantum dots (QDs) gold NPs (AuNP)	specific targeting ability *in vitro*, clear visualization of tumour by SPECT, high accumulation in the liver and the spleen, moderate accumulation in the bone marrow and kidneys, low non-specific tissue uptake and tumour uptake, short blood half-life	[33]

gold NPs (DTDTPA-AuNP)	specific targeting ability, high uptake in the tumour after 24 hours post injection.	[32]
liposomal NPs (LNP)	specific targeting ability *in vitro*, the superiority of J591-radiolabelled liposomes over both A10 PSMA-radiolabelled liposomes in terms of their binding and internalization efficacies, stable encapsulation of 225Ac in liposomes, selective targeting and killing of cell monolayers with variable PSMA expression by J591-targeted and radiolabelled liposomes, targeting and killing of cell monolayers with relatively high PSMA expression by A10-targeted and radiolabelled liposomes.	[34]
liposomal NPs (LNP)	specific targeting ability, similar extents of cellular internalization, delivered radioactivity and cell viability in cells exposed to NPs targeted with J-591 antibody or with the urea-based ligand, perinuclear localization of NPs targeted with the urea-based ligand.	[35]
zeolite NPs	specific targeting ability, high accumulation in the liver, lungs, spleen and bone tissue, marginal accumulation in the tumour, bone marrow fibrosis, decreased the number of WBC and platelets and elevated serum concentrations of ALT and AST enzymes *in vivo*.	[50,59]
curcumin-containing poly(lactic-*co*-glycolic acid) NPs (PLGA-CUR)	specific targeting ability, high accumulation in the tumour and very minimal accumulation in other organs, significantly greater accumulation and retaining within the tumour with NPs-based targeted radioconjugate over radiolabelled antibody, , superior inhibition of tumour growth with NPs-based targeted radioconjugate, compared to curcumin *in vivo.*	[38]
sorafenib -containing silica NPs (PSi)	specific targeting ability, high accumulation in the liver and spleen and minor accumulation in the bladder during the first 1 h, high accumulation and retention in the tumour after intra-tumoral administration, no differences in inhibition of tumour growth by the sorafenib-loaded NPs and free sorafenib,	[60]
micellar NPs (LNP)	specific targeting ability, clear visualization of tumour by PET, rapid accumulation in the liver, tumour and kidney, followed by slow degradation, significant tumour reduction by the mixture of NPs-based radioconjugate with NPs conjugated to monomethyl auristatin E (MMAE, antimicrotubule agent).	[43]
texaphyrin NPs (texaphyrin)	specific targeting ability, high accumulation in the liver and spleen, moderate accumulation in the tumour, followed by hepatobiliary clearance and increasing contrast between the tumour and surrounding organs, a potent effect of photodynamic therapy and successful inhibition of (PSMA+) tumour growth by combination with light irradiation, no adverse effects in major organs	[37]
doxorubicin-containing liposomal NPs modified with P3-liposomes)	specific targeting ability *in vitro*, higher *in vitro* cellular uptake and delivery of doxorubicin into cells by targeted NPs-based radioconjugates than by untargeted NPs-based radioconjugates, higher cytotoxicity of doxorubicin-loaded targeted NPs, compared to doxorubicin-loaded untargeted NPs	[36]
